# Gluten-free diets for metabolic control of type 1 diabetes mellitus in children and adolescents: a systematic review and meta-analysis

**DOI:** 10.20945/2359-4292-2024-0165

**Published:** 2025-03-24

**Authors:** Yan Zhang, Suhong Yang, Pingping Wang

**Affiliations:** 1 Department of Endocrinology, Hangzhou Children’s Hospital, Zhejiang, China

**Keywords:** Gluten-free diet, metabolic control, type 1 diabetes mellitus, children and adolescents, meta-analysis

## Abstract

The aim of this review is to comprehensively assess the association between a gluten-free diet (GFD) and metabolic control of type 1 diabetes mellitus (T1DM) in children and adolescents with T1DM and with T1DM plus coeliac disease (CD). PubMed, Embase, Cochrane Library, and Web of Science were searched until June 19, 2023. Primary outcomes were hemoglobin A1c (HbA1c), insulin dose, insulin dose adjusted A1c (IDAA1c), blood glucose (B-glu) at 90 min during Mixed Meal Tolerance Test (MMTT), C-peptide area under the curve (AUC), and C-peptide. Seven studies involving 355 T1DM patients were included. Three studies involving 141 patients compared a GFD to a standard diet in children and adolescents withT1DM without CD. Additionally, two studies with 164 patients examined the same diet comparison in those with T1DM and concurrent CD. A comparison between T1DM with CD and T1DM alone, using a GFD, was conducted in two studies encompassing 50 patients. Patients withT1DM alone had similar HbA1c [pooled weighted mean difference (WMD) = -0.5, 95% confidence interval (CI): -1.0 to 0.1, *P* = 0.079] and IDAA1c (pooled WMD = -0.4, 95%CI: -0.9 to 0.1, *P* = 0.095) levels after a GFD and a standard diet. In children and adolescents withT1DM and CD, a GFD was associated with a significantly lower HbA1c compared with a standard diet (pooled WMD = -0.64, 95%CI: -1.22 to -0.05, *P* = 0.034). Insulin dose was significantly lower in T1DM combined with CD patients having a GFD *vs* a standard diet (pooled WMD = -0.34, 95%CI: -0.66 to -0.03, *P* = 0.032). Our study suggests that a GFD may offer significant benefits for children and adolescents with both T1DM and CD over a standard diet. While the evidence indicates improved glycemic control with a GFD, the quality of this evidence is low, highlighting the need for rigorous, randomized trials to confirm these preliminary findings. In the interim, enhancing dietary awareness and providing tailored nutritional guidance could be pivotal for optimizing glucose management in this patient population.

## INTRODUCTION

Type 1 diabetes mellitus (T1DM) is a primary type of diabetes and often occurs in the young population with insulin deficiency (^1^). The incidence of T1DM has elevated by 3%-4% in the last three decades (^2^). Celiac disease (CD), or gluten-sensitive enteropathy, is a prevalent genetic autoimmune disorder characterized by small intestinal inflammation triggered by dietary gluten in susceptible individuals (^3^). T1DM and CD are polygenic autoimmune disorders with a high coexistence tendency because of common etiological factors such as genetic and clinicopathological overlaps, and the average prevalence of the coexistence is over 8% (^4^). The prevalence of CD among T1DM children is estimated to range from 1.4% to 19.7% (^5,6,7^).

Currently, the only available treatment for CD is a rigorous gluten-free diet (GFD) through life (^8^). Gluten may be a pathogenic factor in T1DM development (^9^). A study indicates that higher gluten intake during pregnancy is associated with an increased risk of T1DM in offspring (^10^). The introduction of gluten into an infant’s diet either after seven months or before the age of four months is correlated with a heightened likelihood of developing diabetes (^10^). Thereby, several studies have investigated the association between GFD and T1DM. Neuman and cols. (^11^) reported that a GFD kept in the first year following T1DM diagnosis in non-CD children was related to lower hemoglobin A1c (HbA1c) and an extended partial remission period. According to Scaramuzza and cols. (^12^), a GFD may affect glycemic values, HbA1c, insulin requirement, and anthropometric measures such as body mass index (BMI), whereas not all researchers agree on the ultimate impact of a GFD. A prior review suggested that the function of dietary gluten in progression of T1DM and the underlying benefit of a GFD in patients with T1DM remain controversial (^13^). In addition, the association of GFD with T1DM and CD has also been assessed by previous studies. A GFD was shown by Kaukinen and cols. (^14^) to have no influence on the metabolic control of T1DM in patients with CD, whereas a tendency to fewer hypoglycemic episodes and greater glycemic control was observed in patients with T1DM and subclinical CD who received a GFD for one year from a randomized controlled trial (RCT) (^15^). Based on the existing literature, the impact of a GFD on metabolic control of T1DM in children and adolescents with T1DM and with T1DM plus CD is unclear.

This systematic review and meta-analysis aimed to comprehensively assess the association between a GFD and metabolic control of T1DM in children and adolescents with T1DM and with T1DM plus CD.

## METHODS

### Search strategy

PubMed, Embase, Cochrane Library, and Web of Science were comprehensively searched until June 19, 2023. Disagreement was settled via discussion. Medical subject headings (MESH) included “Diabetes Mellitus, Type 1” and “Diet, Gluten-Free”. The search terms used were: “Diet” OR “Gluten-Free” OR “Diet, Gluten Free” OR “Gluten-Free Diet” OR “Diets, Gluten-Free” OR “Gluten Free Diet” OR “Gluten-Free Diets” AND “Diabetes Mellitus, Type 1” OR “Diabetes Mellitus, Insulin-Dependent” OR “Diabetes Mellitus, Insulin Dependent” OR “Insulin-Dependent Diabetes Mellitus” OR “Diabetes Mellitus, Juvenile-Onset” OR “Diabetes Mellitus, Juvenile Onset” OR “Juvenile-Onset Diabetes Mellitus” OR “IDDM” OR “Juvenile-Onset Diabetes” OR “Diabetes, Juvenile-Onset” OR “Juvenile Onset Diabetes” OR “Diabetes Mellitus, Sudden-Onset” OR “Diabetes Mellitus, Sudden Onset” OR “Sudden-Onset Diabetes Mellitus” OR “Type 1 Diabetes Mellitus” OR “Diabetes Mellitus, Insulin-Dependent, 1” OR “Insulin-Dependent Diabetes Mellitus 1” OR “Insulin Dependent Diabetes Mellitus 1” OR “Type 1 Diabetes” OR “Diabetes, Type 1” OR “Diabetes Mellitus, Type I” OR “Diabetes, Autoimmune” OR “Autoimmune Diabetes” OR “Diabetes Mellitus, Brittle” OR “Brittle Diabetes Mellitus” OR “Diabetes Mellitus, Ketosis-Prone” OR “Diabetes Mellitus, Ketosis Prone” OR “Ketosis-Prone Diabetes Mellitus”. For retrieved studies, primary screening was carried out based on titles and abstracts after removing duplicates, following by study selection through full-text reading. This systematic review and meta-analysis was performed following the Preferred Reporting Items for Systematic Reviews and Meta-analyses (PRISMA) reporting guideline (Supplementary Table S1), and was registered in PROSPERO with number CRD42023449506.

### Eligibility criteria

Inclusion criteria were based on the PICOS principles: P (patients): (1) children and adolescents with T1DM with and without CD; (2) I (intervention): GFD; (3) C (comparison): standard diet; (4) O (outcomes): HbA1c, insulin dose, insulin dose adjusted A1c (IDAA1c), blood glucose (B-glu) at 90 min during Mixed Meal Tolerance Test (MMTT), C-peptide area under the curve (AUC), C-peptide, quality of life (QoL), body mass index standard deviation score (BMI SDS), BMI z-score (outcome); (5) S (study design): controlled trials, cohort studies, case-control studies. In the case of studies reporting data from the same population, the latest studies or studies with the most complete data were included.

Exclusion criteria were: (1) studies on animal experiments; (2) conference reports, case reports, editorial materials, letters, protocols, meta-analyses, reviews; (3) studies for which the full text was not available; (4) studies with incomplete data; (5) non-English studies; (6) studies on patients with type 2 diabetes mellitus or aged ≥ 18 years.

### Outcome measures

Primary outcomes were HbA1c (%), insulin dose (U/kg/day), IDAA1c, B-glu at 90 min during MMTT, C-peptide AUC (pmol/L), and C-peptide (pmol/L). Secondary outcomes were QoL, BMI SDS, and BMI z-score.

### Data extraction and quality assessment

Data on first author, year of publication, country, study design, sample size (N), age (years), gender (male/female), duration of T1DM (years), group, intervention time (months), follow-up time (months), quality assessment, and outcome were obtained by two independent authors (JM Zhang, Q Zhou). The Methodological Index for Non-Randomized Studies (MINORS) was applied to assess the quality of non-randomized studies (^16^). There were a total of 12 evaluation items, each with a score of 0 to 2 (0: not reported; 1: reported but inadequate; 2: reported and adequate). For comparative studies, a MINORS score of 7-12 was classified as low quality, 13-18 as medium quality, and 19-24 as high quality (^17^). The quality of case-control and cohort studies was evaluated with the modified Newcastle-Ottawa scale (NOS). The scale had a total score of 9, with 0-3 as low quality, 4-6 as medium quality, and 7-9 as high quality (^18^). The risk of bias in non-randomized studies was assessed using the Cochrane Risk of Bias in Non-Randomised Studies of Interventions (ROBINS-I) tool, and was classified as low, moderate, serious, or critical risk (^19^). The evidence quality for each outcome in this meta-analysis was measured with the Grading of Recommendations Assessment, Development, and Evaluation (GRADE) approach (^20^), and was graded as high, moderate, low or very low.

### Statistical analysis

The included studies were divided into three types to assess the association between a GFD and metabolic control of T1DM in children and adolescents: (1) studies on a GFD *vs.* a standard diet for children and adolescents with T1DM not combined with CD; (2) studies on a GFD *vs.* a standard diet for children and adolescents with T1DM combined with CD; (3) studies on a GFD for children and adolescents with T1DM combined with CD *vs.* T1DM not combined with CD.

For pooled analysis, the effect size of each outcome was tested for heterogeneity. If I^2^ < 50%, the fixed-effects model was selected for analysis, and if I^2^ ≥ 50%, the random-effects model was used for analysis. Separate analysis was carried out for interventional and observational studies. Sensitivity analysis was performed for the outcomes. Since all the data used for analysis were all measurement data, weighted mean differences (WMDs) were utilized as the effect size, which were expressed with 95% confidence intervals (CIs). Forest plots were depicted for pooled results. All studies were statistically analyzed using Stata 15.1 (Stata Corporation, College Station, TX, USA). *P* < 0.05 was deemed significantly different.

## RESULTS

### Characteristics of the included studies

**Figure 1:**
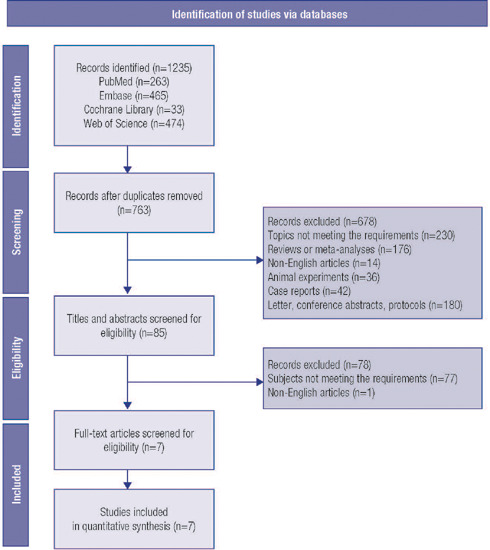
PRISMA flow diagram of study selection.

After searching the four databases, 1,235 studies were identified, with 263 from PubMed, 465 from Embase, 33 from Cochrane Library, and 474 from Web of Science. Then 763 studies left following duplicate removal. In the end, 7 studies (^11,21,22,23,24,25,26^) of 355 T1DM patients were included in this analysis based on screening via titles and abstracts as well as full texts. Figure 1 shows the process of study selection. There were 3 studies of 141 patients on a GFD *vs.* a standard diet for children and adolescents with T1DM not combined with CD, two studies of 164 patients on a GFD *vs.* a standard diet for children and adolescents with T1DM combined with CD, and two studies of 50 patients on a GFD for children and adolescents with T1DM combined with CD *vs.* T1DM not combined with CD. The characteristics of the included studies are presented in Table 1. These included studies included 2 non-randomized controlled studies, 3 case-control studies, and 2 cohort studies. Additionally, 1 study was of low quality, 4 of medium quality, and 2 of high quality. Six studies had a moderate risk of bias, and 1 study had low risk of bias. The outcomes had very low and low evidence quality of evidence due to the low and moderate risk of bias, low sample size, and non-randomized control in the included studies (Supplementary Table S2). The Population, Intervention, Comparator, Outcome, Study Design (PICOS) table of the included studies is exhibited in Table 2.

PRISMA: Preferred Reporting Items for Systematic Reviews and Meta-Analysis.

**Table 1:** Baseline characteristics of the included studies

Author	Year	Country	Study design	N	Age (years)	Gender (M/F)	Duration of T1DM (years)	OG	CG	I time (months)	FU time (months)	QA	Outcome	Risk of bias
**GFD** *vs.* **standard diet for children and adolescents with T1DM not combined with CD**														
Neumann	2020	Czech Republic	Nonrandomized controlled trial	39	10 ± 3.3	OG: 10/10 CG:16/3	-	GFD	Standard diet	12	12	11	C-peptide AUC, HbA1c, IDAAIc, insulin dose	Moderate
Söderström	2022	Sweden	Nonrandomized controlled trial	23	9.3 ±6.83	OG: 8/6 CG: 6/3	-	GFD	Normal diet	12	24	13	HbA1c, IDAA1 c, C-peptide, QoL, B-glu at 90 min during MMTT	Moderate
Simmons	2011	USA	Cohort study	79	10.4 ±0.4	-	3.8 ±0.3	GFD	Regular diet	24	24	8	HbA1c, BMI z-score	Moderate
**GFD *vs.* standard diet for children and adolescents with T1DM combined with CD**														
Pham-Short	2016	Australia	Case-control study	35	OG: 13.5 + 3.1 CG: 14.2 ±3.1	OG: 12/12 CG: 8/3	OG: 7.2 ±3.4 CG: 8.5± 3.0	GFD	Poor compliance GFD	12	18	6	HbA1c, insulin dose, QoL score, BMI z-score	Moderate
Pham-Short	2014	Australia	Case-control study	129	OG: 16.01 ±2.57 CG: 16.19 ± 2.73	-	OG: 9.35± 4.26 CG: 9.33 ±4.56	GFD, adherent	GFD, non-adherent	12	12	8	HbA1c, insulin dose, BMI SDS	Low
**GFD for children and adolescents with T1DM combined with CD** *vs.* **T1DM not combined with CD**														
Amin	2002	UK	Cohort study	33	OG: 8.1 (1.2, 16.1) CG: 7.4(1.3, 14.8)	OG: 5/6 CG: 10/12	OG: 4.2 (0.9-7.2) CG: 4.0 (1.0-10)	CD	Non-CD	12	12	6	HbA1c, insulin dose, C-peptide, BMI SDS	Moderate
Pham-Short	2017	Australia	Case-control study	17	OG: 14.3 ± 3.6 CG: 14.7 ± 2.8	-	OG: 6.7 ±4.0 CG: 5.9 ± 2.2	CD	Non-CD	12	12	5	HbA1c, insulin dose, BMI SDS	Moderate

Age was expressed as mean ± standard deviation or median (interquartile range).

T1DM: type 1 diabetes mellitus: CD: coeliac disease: M/F: male/female: OG: observation group: CG: control group: I time: intervention time: FU time: follow-up time: QA: quality assessment: GFD: gluten-free diet: HbA1c: hemoglobin A1c; IDAA1c: insulin dose adjusted A1c; B-glu at 90 min during MMÎÎ: blood glucose at 90 min during Mixed Meal tolerance lest: C-peptide AUC: C-peptide area under the curve: QoL quality of life: BMI SDS: body mass index standard deviation score.

**Table 2:** PICOS table of the included studies

Author	Study design	Population	Intervention	Comparison	Outcome
Neumann, 2020	Non-randomized controlled trial	39 children with T1DM: 20 GFD subjects and 19 control subjects.	GFD	Standard diet	C-peptide AUC, HbA1c, IDAA1c, insulin dose
Söderström, 2022	Non-randomized controlled trial	Twenty-three children with newly diagnosed T1DM followed a GFD (n = 14) or a normal diet (n = 9) for 12 months.	GFD	Normal diet	HbA1c, IDAA1c, C-peptide, QoL, B-glu at 90 min during MMTT
Simmons, 2011	Cohort study	Children with T1DM: 43 selected to GFD and 36 continue a regular diet.	GFD	Regular diet	HbA1c, BMI z-score
Pham-Short, 2016	Case-control study	Youth with T1DM and CD: 24 of the 35 patients with CD (69%) were classified as GFD+, and 11 of the 35 (31%) as GFD-.	GFD	Poor compliance GFD	HbA1c, insulin dose, QoL score, BMI z-score
Pham-Short, 2014	Case-control study	129 young people with T1DM and coeliac disease: 60 (47%) did not adhere to a gluten-free diet and 69 adhere to a gluten-free diet.	GFD, adherent	GFD, non-adherent	HbA1c, insulin dose, BMI SDS
Amin, 2002	Cohort study	11 children with T1DM and CD on a GFD; 22 Celiac-negative control subjects was matched.	GFD	GFD	HbA1c, insulin dose, C-peptide, BMI SDS
Pham-Short, 2017	Case-control study	10 youth with T1DM and biopsy-proven CD, 10 with T1DM was matched.	GFD	GFD	HbA1c, insulin dose, BMI SDS

HbA1c, insulin dose, BMI SDS

PICOS: Population, Intervention, Comparator, Outcome, Study Design; GFD: gluten-free diet; T1DM: type 1 diabetes mellitus; CD: coeliac disease; SDS: standard deviation score; HbA1c: hemoglobin A1c; IDAA1c: insulin dose adjusted A1c; B-glu at 90 min during MMTT: blood glucose at 90 min during Mixed Meal Tolerance Test; C-peptide AUC: C-peptide area under the curve; QoL: quality of life; BMI: body mass index; SDS: standard deviation score.

### GFD versus standard diet in children and adolescent T1DM without CD

#### HbA1c

**Figure 2:**
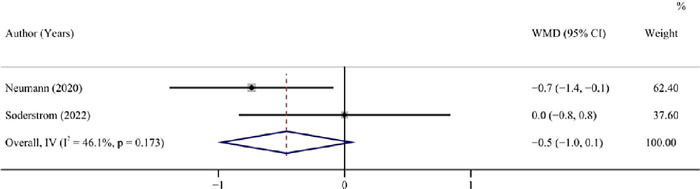
Forest plot for HbA1c after a GFD vs a standard diet in children and adolescents with T1DM not combined with CD.

HbA1c: hemoglobin A1c; GFD: gluten-free diet; T1DM: type 1 diabetes mellitus; CD: coeliac disease; WMD: weighted mean difference; CI: confidence interval.

Three studies (^11,23,24^) including 125 patients provided information on HbA1c, with 2 non-randomized controlled trials (interventional studies), and 1 cohort study (observational study). Pooled analysis of the 2 non-randomized controlled trials showed no significant difference in the HbA1c level between the GFD and standard diet groups (pooled WMD = -0.5, 95%CI: -1.0 to 0.1, I^2^ = 46.10%, *P* = 0.079) (Table 3, Figure 2). Based on the 1 cohort study, the HbA1c levels were similar in the GFD and standard diet groups (WMD = -0.5, 95%CI: -1.0 to 0.0, *P* = 0.054).

**Table 3:** Pooled analysis of GFD for different outcomes in children and adolescents with T1DM

Outcome	Study design	WMD (95%CI)	P	I^2^
GFD vs. standard diet for children and adolescents with T1DM not combined with CD (11,23,24)				
HbA1c (11,23,24)	Interventional	-0.5 (-1.0, 0.1)	0.079	46.10%
IDAA1c (11,23)	Observational	-0.4 (-0.9, 0.1)	0.095	0.00%
GFD vs. standard diet for children and adolescents with T1DM combined with CD (25,26)				
HbA1c (25,26)	Observational	-0.64 (-1.22, -0.05)	0.034	54.30%
Insulin dose (25,26)	Observational	-0.34 (-0.66, -0.03)	0.032	9.10%
GFD on outcomes in children and adolescents with T1DM combined with CD vs. children and adolescents with T1DM not combined with CD (21,22)				
HbA1c (21,22)	Observational	-4.5 (-12.3, 3.4)	0.263	97.50%
Insulin dose (21,22)	Observational	0.1 (-0.5, 0.7)	0.751	0.00%
BMI SDS (21,22)	Observational	0.4 (-0.8, 1.6)	0.488	73.60%

GFD: gluten-free diet; T1DM: type 1 diabetes mellitus; CD: coeliac disease; HbA1c: hemoglobin A1c; IDAA1c: insulin dose adjusted A1c; BMI SDS: body mass index standard deviation score; WMD: weighted mean difference; CI: confidence interval.

#### Insulin dose

One study (^11^) with 39 patients illustrated that the insulin dose was significantly lower after a GFD a standard diet (WMD = -0.9, 95%CI: -1.5 to -0.2, *P* = 0.009).

#### IDAA1c

**Figure 3:**
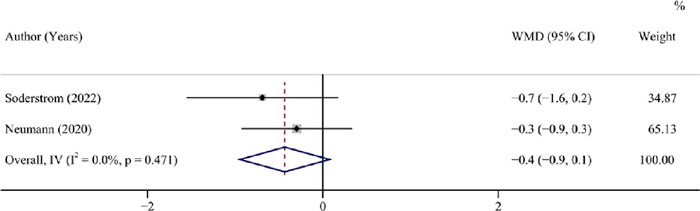
Forest plot for IDAA1c after a GFD *vs* a standard diet in children and adolescents with T1DM not combined with CD.

IDAA1c: insulin dose adjusted A1c; GFD: gluten-free diet; T1DM: type 1 diabetes mellitus; CD: coeliac disease; WMD: weighted mean difference; CI: confidence interval.

Patients with a GFD had a comparable level of IDAA1c to those with a standard diet, according to two studies (^11,23^) with 62 patients (pooled WMD = -0.4, 95%CI: -0.9 to 0.1, I^2^ = 0.00°%, *P* = 0.095) (Table 3, Figure 3).

#### B-glu at 90 min during MMTT

One study (^23^) with 23 patients showed that a GFD was associated with a similar level of B-glu at 90 min during MMTT to a standard diet (WMD = -0.4, 95%CI: -1.3 to 0.4, *P* = 0.327).

#### C-peptide AUC

No significant difference was found in C-peptide AUC between patients receiving a GFD and a standard diet, based on 1 study (^11^) with 39 patients (WMD = -0.1, 95%CI: -0.7 to 0.6, *P* = 0.813).

#### C-peptide

A study (^23^) with 23 patients exhibited equivalent levels of C-peptide in patients who had GFD and a standard diet (WMD = -0.4, 95%CI: -1.2 to 0.5, *P* = 0.396).

#### QoL

In accordance with 1 study (^23^) of 23 patients, diabetes-related problems with QoL were similar after a GFD and a standard diet (WMD = 0.7, 95%CI: -0.1 to 1.6, *P* = 0.091).

#### BMI z-score

Based on one study (^24^) of 63 patients, patients with a GFD exhibited a significantly lower BMI z-score than those having a standard diet (WMD = -2.3, 95%CI: -2.9 to -1.6, *P* < 0.001).

### GFD versus standard diet in children and adolescent T1DM combined with CD

#### HbA1c

**Figure 4:**
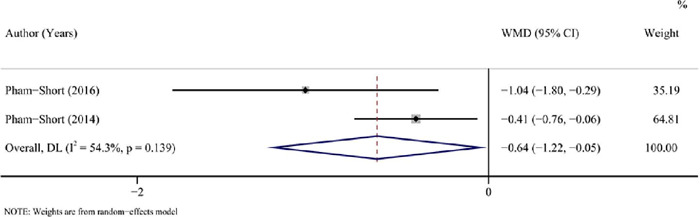
Forest plot for HbA1c after a GFD *vs* a standard diet in children and adolescents with T1DM combined with CD.

HbA1c: hemoglobin A1c; GFD: gluten-free diet; T1DM: type 1 diabetes mellitus; CD: coeliac disease; WMD: weighted mean difference; CI: confidence interval.

Patients with a GFD had a significantly lower HbA1c compared with those with a standard diet, as comprehensively assessed by 2 studies (^25,26^) with 164 patients (pooled WMD = -0.64, 95%CI: -1.22 to -0.05, I^2^ = 54.30%, *P* = 0.034) (Table 3, Figure 4).

#### Insulin dose

**Figure 5:**
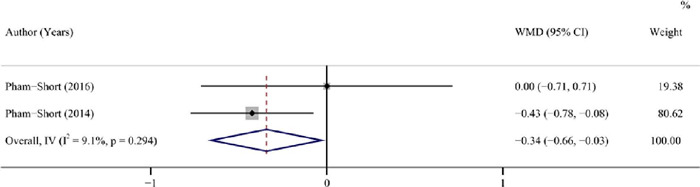
Forest plot for insulin dose after a GFD *vs* a standard diet in children and adolescents with T1DM combined with CD.

GFD: gluten-free diet; T1DM: type 1 diabetes mellitus; CD: coeliac disease; WMD: weighted mean difference; CI: confidence interval.

Two studies (^25,26^) with 164 patients showed that insulin dose was significantly lower in patients having a GFD a standard diet (pooled WMD = -0.34, 95%CI: -0.66 to -0.03, I^2^ = 9.10%, *P* = 0.032) (Table 3, Figure 5).

#### BMI z-score

One study (^25^) of 35 patients demonstrated that patients having a GFD and a standard diet had similar BMI z-scores (WMD = -0.3, 95%CI: -1.0 to 0.5, *P* = 0.478).

#### BMI SDS

Patients with a GFD were illustrate by 1 study (^26^) with 129 patients to have a comparable BMI SDS to those with a standard diet (WMD = -0.33, 95%CI: -0.68 to 0.02, *P* = 0.061).

### GFD in children and adolescent T1DM with and without CD

#### HbA1c

**Figure 6:**
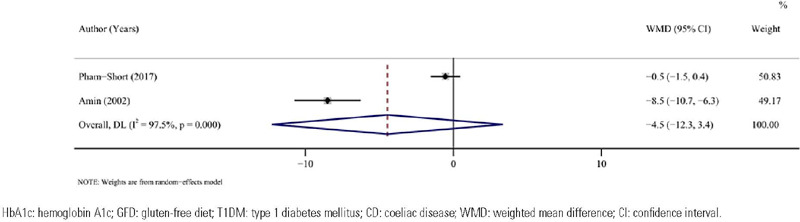
Forest plot for HbA1c after a GFD in children and adolescents with T1DM combined with CD *vs* T1DM not combined with CD.

HbA1c: hemoglobin A1c; GFD: gluten-free diet; T1DM: type 1 diabetes mellitus; CD: coeliac disease; WMD: weighted mean difference; CI: confidence interval.

Assessment of HbA1c was conducted in 2 studies (^21,22^) with 50 patients. Combined analysis demonstrated that HbA1c in patients with T1DM combined with CD was equivalent to that in patients with T1DM not combined with CD under a GFD (pooled WMD = -4.5, 95%CI: -12.3 to 3.4, I^2^ = 97.5%, *P* = 0.263) (Table 3, Figure 6).

#### Insulin dose

**Figure 7:**
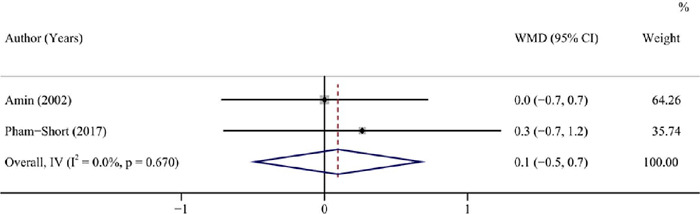
Forest plot for insulin dose after a GFD in children and adolescents with T1DM combined with CD *vs* T1DM not combined with CD.

GFD: gluten-free diet; T1DM: type 1 diabetes mellitus; CD: coeliac disease; WMD: weighted mean difference; CI: confidence interval.

Pooled analysis of 2 studies (^21,22^) with 50 patients exhibited similar insulin dose among patients with T1DM combined with and not combined with CD when having a GFD (pooled WMD = 0.1, 95%CI: -0.5 to 0.7, I^2^ = 0.00%, *P* = 0.751) (Table 3, Figure 7).

#### C-peptide

As found by Amin and cols. (^21^) in 33 patients, there was no significant difference in the C-peptide level after a GFD between patients with T1DM combined with and not combined with CD (WMD = -0.2, 95%CI: -0.9 to 0.5, *P* = 0.597).

#### BMI SDS

**Figure 8:**
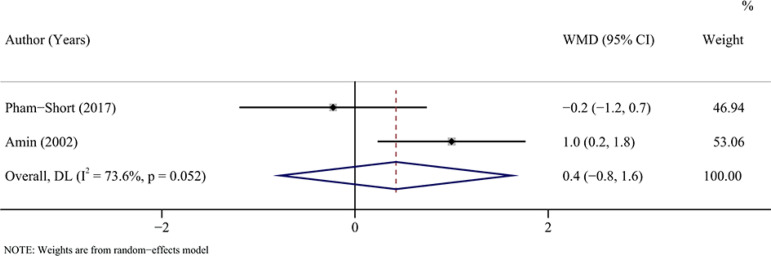
Forest plot for BMI SDS after a GFD in children and adolescents with T1DM combined with CD *vs* T1DM not combined with CD.

Based on two studies (^21,22^) with 50 patients, no significant difference was observed in the BMI SDS between patients with T1DM combined with and not combined with CD who had a GFD (pooled WMD = 0.4, 95%CI: -0.8 to 1.6, I^2^ = 73.60%, *P* = 0.488) (Table 3, Figure 8).

BMI SDS: body mass index standard deviation score; GFD: gluten-free diet; T1DM: type 1 diabetes mellitus; CD: coeliac disease; WMD: weighted mean difference; CI: confidence interval.

#### Sensitivity analysis

Sensitivity analysis was performed through removal of a study at a time and comprehensively analyzing the remaining studies. It was demonstrated that one-study removal did not significantly affect the combined results, suggesting the consistency of the findings of the meta-analyses.

## DISCUSSION

The present systematic review with meta-analysis shows that in children and adolescents with T1DM comparable HbA1c and IDAA1c levels were observed following a GFD or a standard diet. However, in children and adolescents with T1DM and CD on a GFD was associated with lower HbA1c levels and insulin dosages than those with a standard diet. Notable, HbA1c levels and insulin doses were similar in children and adolescents with T1DM and CD in comparison with T1DM alone under a GFD. To our knowledge, this meta-analysis was the first to comprehensively analyze the association between a GFD and metabolic control in children and adolescents with T1DM as well as with T1DM plus CD, as previous systematic reviews focused on children and adolescents solely with the combination of T1DM and CD.

Burayzat and cols. (^27^) performed a meta-analysis of case-control studies to assess whether a GFD affected BMI and HbA1c in children and adolescents with T1DM and symptomless CD. They found that a GFD exerted no significant influence on BMI or HbA1c. A recent review by Mozzillo and cols. (^28^) included RCTs, observational studies, exploratory studies, mix of qualitative and quantitative studies to evaluate the effect of a GFD on growth, metabolic control and QoL in children and adolescents with T1DM and CD, and indicated that adherence to a GFD resulted in normal growth, steady BMI, and improved QoL without any adverse impact on HbA1c and insulin needs. The current study focused on children and adolescents with T1DM and children and adolescents with T1DM and CD, respectively. The difference among these studies is the different designs of studies included and different study groups.

Prior evidence illustrated that removing gluten from diets could selectively prevent the progression of diabetes (^29,30^). In this analysis, similar HbA1c and IDAA1c levels were exhibited in T1DM patients having a GFD and a standard diet, which may be attributed to small sample sizes. Future large-scale studies are warranted to verify the relationship between GFDs and HbA1c levels in T1DM. In this study, for children and adolescents with T1DM and CD, the HbA1c level and insulin dose following a GFD were lower than those after a standard diet, suggesting better glycemic control under a GFD. Eland and cols. (^31^) reported the benefits of GFDs for HbA1c levels and insulin requirements in individuals with both T1DM and CD. Diets without gluten may affect insulin sensitivity, which may be a reason for positive results concerning the HbA1c and insulin dose (^32^). Besides, some beneficial impacts of a GFD may explain the improved HbA1c and insulin dose after a GFD. Gluten can increase intestinal permeability, and elevated permeability enables macromolecules to enter the bloodstream from the intestine and possibly induces generation of many pro-inflammatory cytokines including IFN-γ, TNF-α and IL-17 (^33,34^). For another, a GFD alters intestinal microbiota composition (^30^). Increased *Akkermansia muciniphil,* which provides protection from T1DM, consumes the mucus layer in the intestinal tract, resulting in great mucin synthesis and tight junction, thereby improving intestinal integrity (^35^). In addition, we found that HbA1c levels and insulin doses were comparable in children and adolescents with T1DM and CD and with T1DM alone under a GFD, suggesting that a GFD may exerts similar influences in these two population. It is important to consider that the improvement in glycemic control observed in the T1DM and CD population may be attributed to the treatment of CD, which could enhance overall metabolism and glycemic management (^36^). Our findings underscore that for individuals with both T1DM and CD, close monitoring and regular consultations with healthcare providers are essential. These individuals may need to adjust their insulin regimen and dietary plans to accommodate the changes brought by a gluten-free diet.

However, a strict GFD can result in deficiencies of fibers as GFD are generally very low in fiber (^37^). Fiber has a significant effect on improving glycemic control (^38^). A systematic review and meta-analysis has identified that a high-fiber diet is an integral component of diabetes management, capable of improving glycemic control (^39^). Large-scale prospective cohort studies consistently demonstrate that, after adjusting for confounding factors, a high intake of dietary fiber is associated with a 20%-30% reduction in the risk of developing type 2 diabetes (^40^). It may be important for healthcare providers to consider strategies to ensure that children and adolescents with T1DM and CD on a GFD still receive adequate dietary fiber.

This study suggested that children and adolescents with T1DM and CD may get better metabolic control of T1DM through a GFD. Greater dietary awareness, closer monitoring of dietary intake and glucose metabolism, professional guidance of dietitians may facilitate management of T1DM in young patients. There were several limitations in this study. First, only English studies were included, which may cause language bias. Second, the results of pooled analysis may be unstable and biased due to limited studies and sample sizes included in the current meta-analysis and very low and low evidence quality of evidence for the outcomes, and more large-scale, high-quality investigations are necessitated to improve the comprehensive assessment of the relationship between GFDs and metabolic control of T1DM in children and adolescents. Third, some outcomes such as C-peptide AUC and B-glu at 90 min during MMTT were only evaluated in one study, and qualitative analysis was carried out. Fourth, the findings were primarily based on observational data, which were inherently subject to various biases that may influence the results and limit the ability to establish causality. The reliance on non-randomized controlled studies further compounded the potential for selection bias and other confounding factors, reducing the strength of conclusions that can be drawn from the data.

In conclusion, the systematic review and meta-analysis suggest that children and adolescents with T1DM and CD who adhere to a GFD may experience lower HbA1c levels and reduced insulin dosages compared to those following a standard diet. However, given the observational nature of the data and the lack of large randomized controlled trials, these findings should be interpreted with caution. The quality of evidence for the reported outcomes is currently very low to low, underscoring the need for higher quality studies to validate these preliminary results. Future research, particularly large-scale randomized clinical trials, is warranted to confirm the potential benefits of a GFD in glycemic control for this population and to provide more definitive guidance for clinical practice.

## SUPPLEMENTARY MATERIAL

**Supplementary Table S1:** PRISMA 2020 checklist

Section and Topic	Item #	Checklist item	Location where item is reported
**TITLE**			
Title	1	Identify the report as a systematic review.	Page 1
**ABSTRACT**			
Abstract	2	See the PRISMA 2020 for Abstracts checklist.	Page 1-2
**INTRODUCTION**			
Rationale	3	Describe the rationale for the review in the context of existing knowledge.	Page 3-4
Objectives	4	Provide an explicit statement of the objective(s) or question(s) the review addresses.	Page 4
**METHODS**			
Eligibility criteria	5	Specify the inclusion and exclusion criteria for the review and how studies were grouped for the syntheses.	Page 5-6
Information sources	6	Specify all databases, registers, websites, organisations, reference lists and other sources searched or consulted to identify studies. Specify the date when each source was last searched or consulted.	Page 4-5
Search strategy	7	Present the full search strategies for all databases, registers and websites, including any filters and limits used.	Page 4-5
Selection process	8	Specify the methods used to decide whether a study met the inclusion criteria of the review, including how many reviewers screened each record and each report retrieved, whether they worked independently, and if applicable, details of automation tools used in the process.	Page 5-6
Data collection process	9	Specify the methods used to collect data from reports, including how many reviewers collected data from each report, whether they worked independently, any processes for obtaining or confirming data from study investigators, and if applicable, details of automation tools used in the process.	Page 6
Data items	10a	List and define all outcomes for which data were sought. Specify whether all results that were compatible with each outcome domain in each study were sought (e.g. for all measures, time points, analyses), and if not, the methods used to decide which results to collect.	Page 7
	10b	List and define all other variables for which data were sought (e.g. participant and intervention characteristics, funding sources). Describe any assumptions made about any missing or unclear information.	Page 7
Study risk of bias assessment	11	Specify the methods used to assess risk of bias in the included studies, including details of the tool(s) used, how many reviewers assessed each study and whether they worked independently, and if applicable, details of automation tools used in the process.	Page 8
Effect measures	12	Specify for each outcome the effect measure(s) (e.g. risk ratio, mean difference) used in the synthesis or presentation of results.	Page 8
Synthesis methods	13a	Describe the processes used to decide which studies were eligible for each synthesis (e.g. tabulating the study intervention characteristics and comparing against the planned groups for each synthesis (item #5)).	Page 8
	13b	Describe any methods required to prepare the data for presentation or synthesis, such as handling of missing summary statistics, or data conversions.	Page 7-8
	13c	Describe any methods used to tabulate or visually display results of individual studies and syntheses.	Page 8
	13d	Describe any methods used to synthesize results and provide a rationale for the choice(s). If meta-analysis was performed, describe the model(s), method(s) to identify the presence and extent of statistical heterogeneity, and software package(s) used.	Page 7-8
	13e	Describe any methods used to explore possible causes of heterogeneity among study results (e.g. subgroup analysis, meta-regression).	Page 7-8
	13f	Describe any sensitivity analyses conducted to assess robustness of the synthesized results.	Page 7-8
Reporting bias assessment	14	Describe any methods used to assess risk of bias due to missing results in a synthesis (arising from reporting biases).	Page 8
Certainty assessment	15	Describe any methods used to assess certainty (or confidence) in the body of evidence for an outcome.	Page 8
**RESULTS**			
Study selection	16a	Describe the results of the search and selection process, from the number of records identified in the search to the number of studies included in the review, ideally using a flow diagram.	Page 8
	16b	Cite studies that might appear to meet the inclusion criteria, but which were excluded, and explain why they were excluded.	Page 8
Study characteristics	17	Cite each included study and present its characteristics.	Page 8-9
Risk of bias in studies	18	Present assessments of risk of bias for each included study.	Page 8-9
Results of individual studies	19	For all outcomes, present, for each study: (a) summary statistics for each group (where appropriate) and (b) an effect estimate and its precision (e.g. confidence/credible interval), ideally using structured tables or plots.	Page 9-12
Results of syntheses	20a	For each synthesis, briefly summarise the characteristics and risk of bias among contributing studies.	Page 9-12
	20b	Present results of all statistical syntheses conducted. If meta-analysis was done, present for each the summary estimate and its precision (e.g. confidence/credible interval) and measures of statistical heterogeneity. If comparing groups, describe the direction of the effect.	Page 9-12
	20c	Present results of all investigations of possible causes of heterogeneity among study results.	Page 9-12
	20d	Present results of all sensitivity analyses conducted to assess the robustness of the synthesized results.	Page 12
Reporting biases	21	Present assessments of risk of bias due to missing results (arising from reporting biases) for each synthesis assessed.	Page 9
Certainty of evidence	22	Present assessments of certainty (or confidence) in the body of evidence for each outcome assessed.	Page 9-12
**DISCUSSION**			
Discussion	23a	Provide a general interpretation of the results in the context of other evidence.	Page 12-13
	23b	Discuss any limitations of the evidence included in the review.	Page 15
	23c	Discuss any limitations of the review processes used.	Page 15-16
	23d	Discuss implications of the results for practice, policy, and future research.	Page 16
**OTHER INFORMATION**			
Registration and protocol	24a	Provide registration information for the review, including register name and registration number, or state that the review was not registered.	NA
	24b	Indicate where the review protocol can be accessed, or state that a protocol was not prepared.	NA
	24c	Describe and explain any amendments to information provided at registration or in the protocol.	NA
Support	25	Describe sources of financial or non-financial support for the review, and the role of the funders or sponsors in the review.	Page 16
Competing interests	26	Declare any competing interests of review authors.	Page 16
Availability of data, code and other materials	27	Report which of the following are publicly available and where they can be found: template data collection forms; data extracted from included studies; data used for all analyses; analytic code; any other materials used in the review.	Page 16

From: Page MJ, McKenzie JE, Bossuyt PM, Boutron I, Hoffmann TC, Mulrow CD, et al. The PRISMA 2020 statement: an updated guideline for reporting systematic reviews. BMJ 2021;372:n71. doi: 10.1136/bmj.n71

For more information, visit: http://www.prisma-statement.org/

**Supplementary Table S2:** GRADE assessment of evidence quality for each outcome

		Certainty assessment				No. of patients			Effect		Certainty	Importance
N° of studies	Study design	Risk of bias	Inconsistency	Indirectness	Imprecision	Other considerations	GFD	Normal diet	Relative (95% CI)	Absolute (95% CI)		
HbA1c(%)-T1DM												
2	Non-randomized trials	Serious^a^	Not serious	Not serious	Serious^b,c^	None	31	28	-	MD 0.5 lower (1.0 lower to 0.1 higher)	⨁⨁○○ Low	CRITICAL
HbA1c (%)-T1DM												
1	Observational studies	Serious^a^	Not serious	Not serious	Serious^b^	None	3/	26	-	MD 0.5 lower (1.0 lower to 0.0 higher)	⨁○○○ Very low	CRITICAL
IDAA1C-T1DM												
2	Non-randomized trials	Serious^a^	Not serious	Not serious	Serious^b,c^	None	31	28	-	MD 0.4 lower (0.9 lower to 0.1 higher)	⨁⨁○○ Low	CRITICAL
HbA1c (%)-T1DM+CD												
2	Observational studies	Serious^a^	Not serious	Not serious	Serious^b^	None	93 cases 71 controls - 0.0%		-0.61 -(-1.22 to-0.05)	-	⨁○○○ Very low	CRITICAL
Insulin dose-T1DM+CD												
2	Observational studies	Serious^a^	Not serious	Not serious	Serious^b^	None	93 cases 71 controls - 0.0%		-0.34 -(-0.66 to-0.03)	-	⨁○○○ Very low	CRITICAL
HbA1c (%)-T1DM+CD *VS.* T1DM												
2	Observational studies	Serious^a^	Not serious	Not serious	Serious^b^	None	71	79	-	MD 4.5 lower (12.3 lower to 3.1 higher)	⨁○○○ Very low	CRITICAL
Insulin dose-T1DM+CD *VS.* T1DM												
2	Observational studies	Serious^a^	Not serious	Not serious	Serious^b^	None	21	29	-	MD 0.1 higher (0.5 lower to 0.7 higher)	⨁○○○ Very low	CRITICAL
BMI SDS-T1DM+CD *VS.* T1DM												
2	Observational studies	Serious^a^	Not serious	Not serious	Serious^b^	None	21	29	-	MD 0.4 higher (0.8 lower to 1.6 higher)	⨁○○○ Very low	IMPORTANT
Insulin dose-T1DM												
1	Non-randomized trials	Serious^a^	Not serious	Not serious	Serious^b,c^	None	20	19	-	MD 0.9 lower (1.5 lower to 0.2 lower)	○○○○ Low	IMPORTANT
Q0I-T1DM												
1	Non-randomized trials	Serious^a^	Not serious	Not serious	Serious^b,c^	None	20	19	-	MD 0.7 higher (0.1 lower to 1.6 higher)	⨁⨁○○ Low	NOT IMPORTANT
C-peptide AUC (pmol/L)-T1DM												
1	Nonrandomized trials	Serious^a^	Not serious	Not serious	Serious^b,c^	None	2D	19	-	MD 0.1 lower (0.7 lower to 0.6 higher)	⨁⨁○○ Low	IMPORTANT
C peptide (pmol/L)-T1DM												
1	Non-randomized trials	Serious^a^	Not serious	Not serious	Serious^b,c^	None	**11**	**9**	-	MD 0.4 lower (1.2 lower to 0.5 higher)	⨁⨁○○ Low	NOT IMPORTANT
B-glu at 90 min during MMTT-T1DM												
1	Non-randomized trials	Serious^a^	Not serious	Not serious	Serious^b,c^	None	11	9	-	MD 0.4 lower (1.3 lower to 0.4 higher)	⨁⨁○○ Low	IMPORTANT
BMI z-score-T1DM												
1	Observational studies	Serious^a^	Not serious	Not serious	Serious^b^	None	3/	26	-	MD 2.3 lower (2.9 lower to 1.6 lower)	⨁○○○ Very low	NOT IMPORTANT
BMI SDS-T1DM+CD												
1	Observational studies	Not serious	Not serious	Not serious	Serious^b^	None	69 cases 60 controls - 0.0%		-0.33 -(-0.68 to 0.02)	-	⨁○○○ Very low	NOT IMPORTANT
BMI z-score-T1DM+CD												
1	Observational studies	Serious^a^	Not serious	Not serious	Serious^b^	None	24 cases 11 controls - 0.0%		-0.3 -(-1.0 to 0.5)	-	⨁○○○ Very low	NOT IMPORTANT
C-peptide (pmol/L)-T1DM+CD *VS.* T1DM												
1	Observational studies	Serious^a^	Not serious	Not soumis	Serious^b^	None	11	77	-	MD 0.2 lower (0.9 lower to 0.5 higher)	○○○○ Very low	IMPORTANT

^a^ROBINS-1 results of some literature: moderate.^b^ Low sample size.^c^ Non-randomized control. GRADE, Grading of Recommendations Assessment, Development, and Evaluation; CI, confidence interval; MD, mean difference; GFD, gluten-free diet; T1DM, type 1 diabetes mellitus; CD, coeliac disease; HbA1c, hemoglobin A1c; IDAA1C, insulin dose adjusted A1c; B-glu at 90 min during MMTT, blood glucose at 90 min during Mixed Meal Tolerance Test; C-peptide AUC, C-peptide area under the curve; QoL, quality of life; BMI, body mass index; SDS, standard deviation score; ROBINS-1, Cochrane Risk of Bias in Non-Randomised Studies of Interventions.

Copyright© *AE&M* all rights reserved.
